# Supplementary figures and images for: MULT: An allometric body mass index (ABMI) reference to assess nutritional status of multiethnic children and adolescents
Source: PLoS One. 2024 Sep 12;19(9):e0305790. doi: 10.1371/journal.pone.0305790 (PMC11392350; doi:10.1371/journal.pone.0305790)

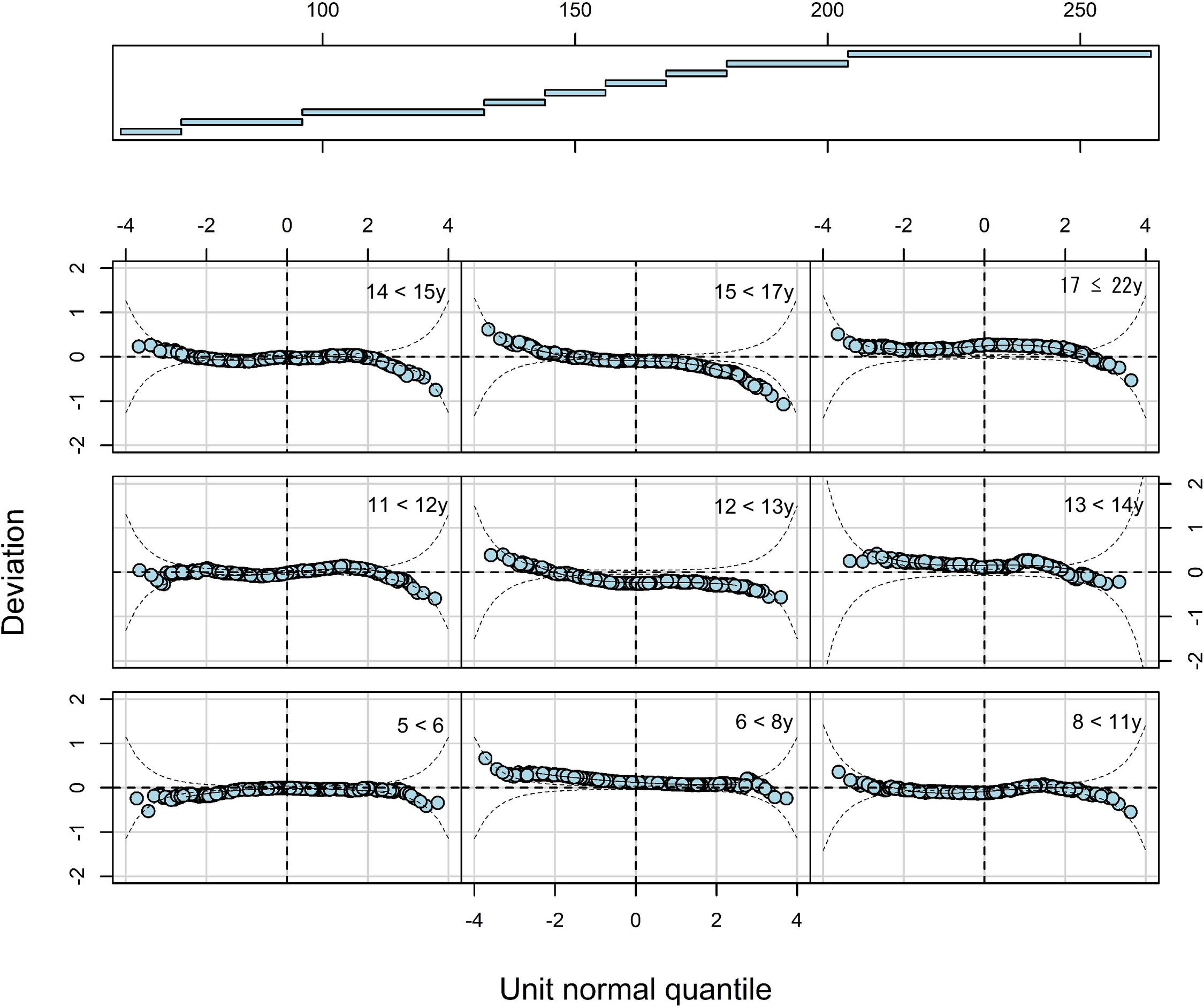

Supplement: S1 Fig — (TIF) [file pone.0305790.s001.tif]

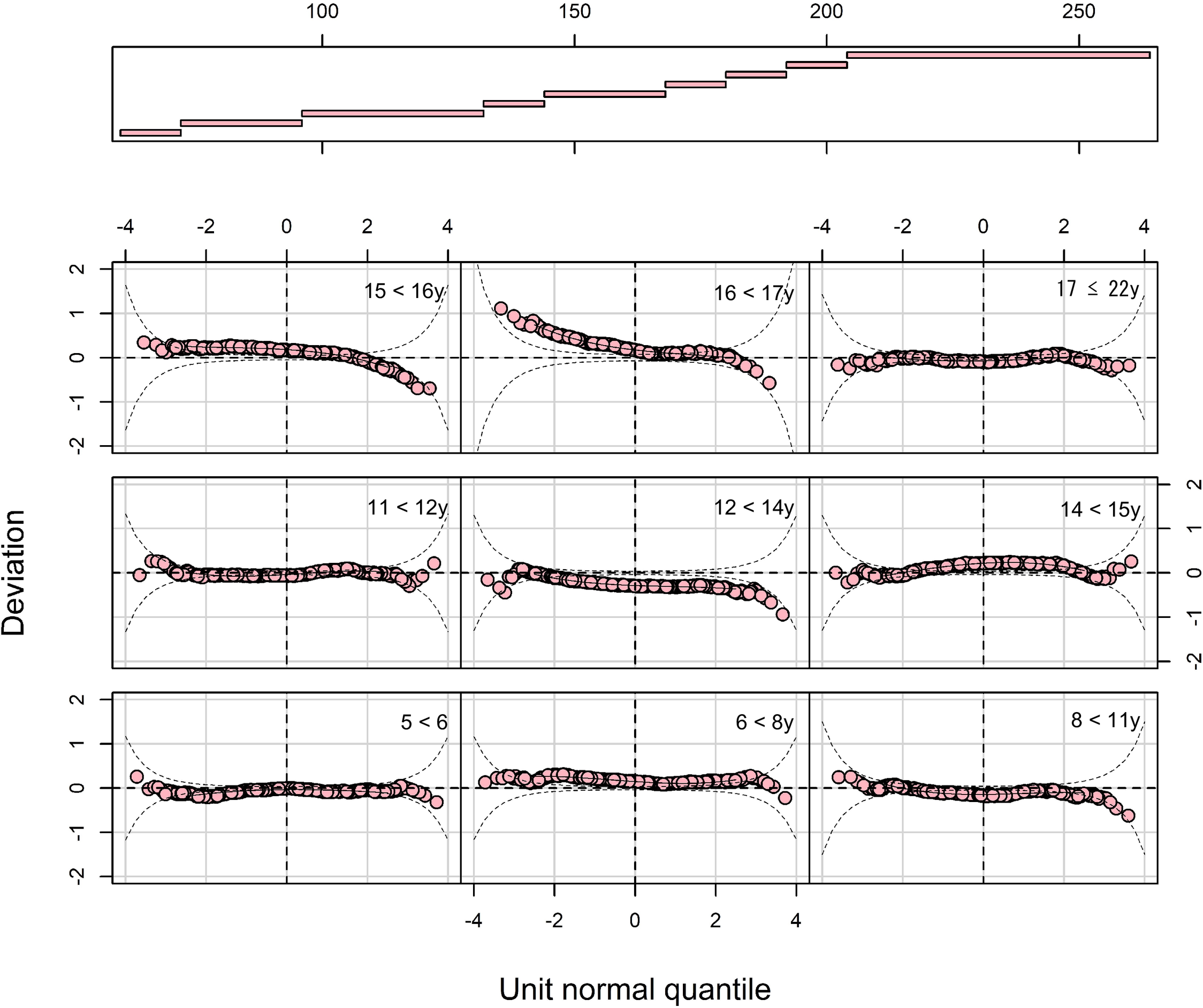

Supplement: S2 Fig — (TIF) [file pone.0305790.s002.tif]

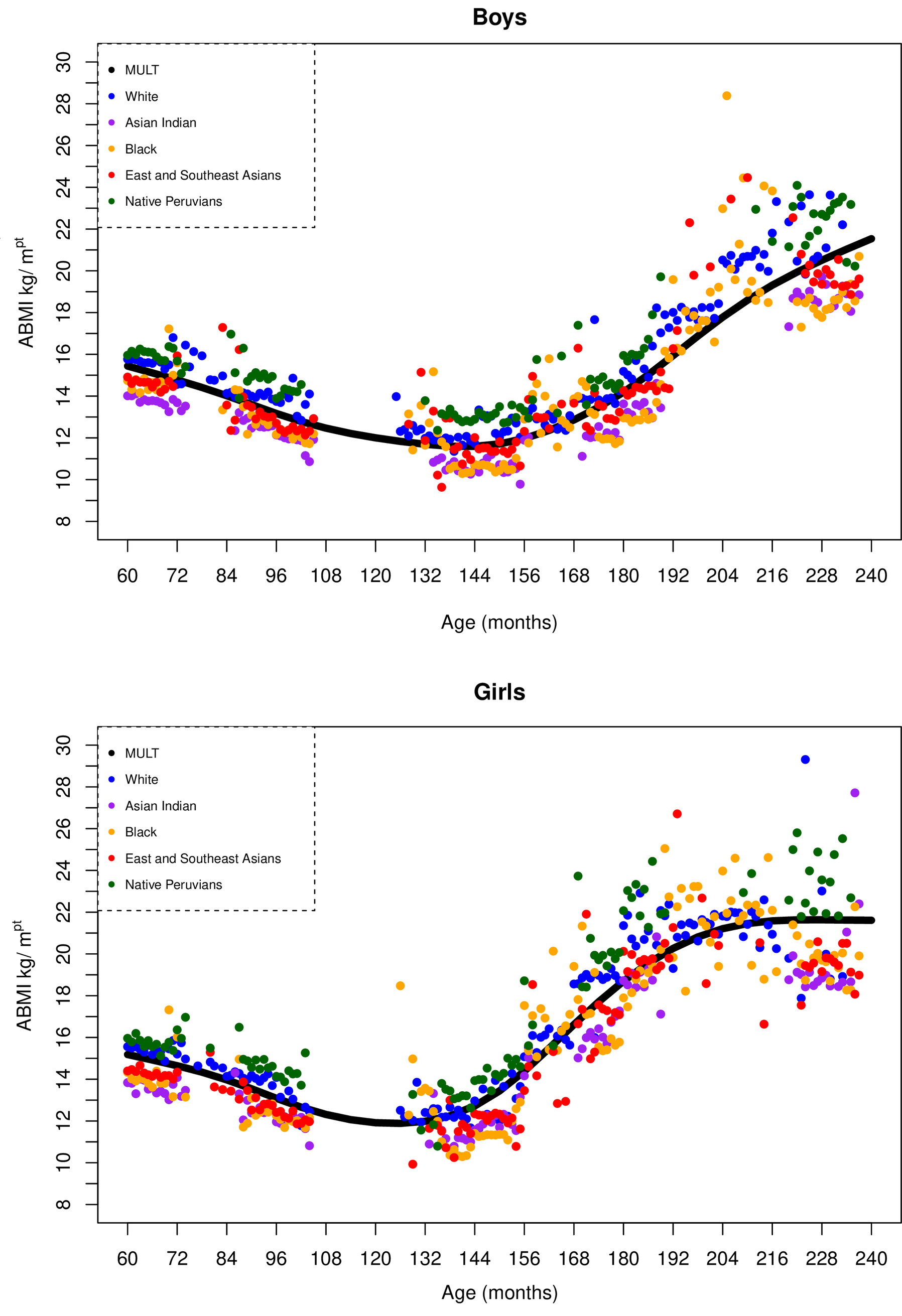

Supplement: S3 Fig — (TIF) [file pone.0305790.s003.tif]
